# Repeated Recruitment of LTR Retrotransposons as Promoters by the Anti-Apoptotic Locus *NAIP* during Mammalian Evolution

**DOI:** 10.1371/journal.pgen.0030010

**Published:** 2007-01-12

**Authors:** Mark T Romanish, Wynne M Lock, Louie N. van de Lagemaat, Catherine A Dunn, Dixie L Mager

**Affiliations:** 1 Terry Fox Laboratory, British Columbia Cancer Agency, Vancouver, British Columbia, Canada; 2 Department of Medical Genetics, University of British Columbia, Vancouver, British Columbia, Canada; Fred Hutchinson Cancer Research Center, United States of America

## Abstract

Neuronal apoptosis inhibitory protein (NAIP, also known as BIRC1) is a member of the conserved inhibitor of apoptosis protein (IAP) family. Lineage-specific rearrangements and expansions of this locus have yielded different copy numbers among primates and rodents, with human retaining a single functional copy and mouse possessing several copies, depending on the strain. Roles for this gene in disease have been documented, but little is known about transcriptional regulation of *NAIP.* We show here that *NAIP* has multiple promoters sharing no similarity between human and rodents. Moreover, we demonstrate that multiple, domesticated long terminal repeats (LTRs) of endogenous retroviral elements provide *NAIP* promoter function in human, mouse, and rat. In human, an LTR serves as a tissue-specific promoter, active primarily in testis. However, in rodents, our evidence indicates that an ancestral LTR common to all rodent genes is the major, constitutive promoter for these genes, and that a second LTR found in two of the mouse genes is a minor promoter. Thus, independently acquired LTRs have assumed regulatory roles for orthologous genes, a remarkable evolutionary scenario. We also demonstrate that 5′ flanking regions of IAP family genes as a group, in both human and mouse are enriched for LTR insertions compared to average genes. We propose several potential explanations for these findings, including a hypothesis that recruitment of LTRs near *NAIP* or other IAP genes may represent a host-cell adaptation to modulate apoptotic responses.

## Author Summary

When retroviruses infect cells, the viral DNA inserts into the cellular genome. If this happens in gametes (egg or sperm), the viral DNA will be transmitted from parent to offspring, like all chromosomal DNA. Through evolutionary time, such infections of gametes have been so prevalent that 8%–10% of the normal human and mouse genomes are now composed of ancient viral DNA, termed endogenous retroviruses (ERVs). In human, these ERVs are mutated or “dead” but it has been shown that ERV regulatory regions can be employed by the host to help control expression of cellular genes. Here, we report on a remarkable example of this phenomenon. We demonstrate that both the human and rodent neuronal apoptosis inhibitory protein *(NAIP)* genes, involved in preventing cell death, use different ERV sequences to drive gene expression. Moreover, in each of the primate and rodent lineages, two separate ERVs contribute to *NAIP* gene expression. This repeated ERV recruitment by *NAIP* genes throughout evolution is very unlikely to have occurred by chance. We offer a number of potential explanations, including the intriguing possibility that it may be advantageous for anti-cell death genes like *NAIP* to use ERVs to control their expression. These results support the view that not all retroviral remnants in our genome are simply junk DNA.

## Introduction

The prevalence of transposed elements (TEs) in mammalian genomes is now well documented [1,2], and their inclusion within human and mouse transcription units is not uncommon. While relatively few genes adopt TEs in their coding regions, primarily as alternative exons recruited from introns [3,4], ∼25% of genes incorporate these sequences into their promoter [5] and UTRs [6]. Moreover, host recruitment of endogenous retrovirus (ERV) long terminal repeats (LTRs), as alternative gene promoters due to their strong RNA polymerase II regulatory signals, is becoming better recognized [7,8]. Whether by altering a protein's conformation, contributing to UTR structure, or donating regulatory signals, LTRs and other TEs can catalyze evolution of new functions or expression patterns of existing genes [8–10]. Furthermore, ERV proteins themselves can participate in important host functions. For example, it appears that independently acquired ERV *env* genes in different mammals have assumed convergent roles in placental development [11,12].

This study documents an extremely unusual case of LTR-mediated transcriptional regulation involving the mammalian neuronal apoptosis inhibitory protein *(NAIP;* also termed *BIRC1)* genes. NAIP belongs to the inhibitor of apoptosis protein (IAP) family, with all members sharing an N-terminal baculoviral IAP repeat (BIR) domain responsible for sequestering activated caspases [13]. The C-termini of individual IAPs, however, are more variable in terms of domain composition, permitting specialization in protein function [14]. Based on its central nucleotide-binding site (NBS) and C-terminal leucine rich repeat (LRR), NAIP is also included in the CATERPILLAR family of proteins, required in the mammalian innate immune response [15]. (Please note that use of the term IAP here is unrelated to the intracisternal A-type particle [IAP] family of mouse retroviral elements [16], which also possess their own LTRs.)

Of the eight orthologous human and mouse IAP family members, *NAIP* has undergone the most extensive genomic rearrangements during mammalian evolution. Human *NAIP* lies within a tract of four other genes that have undergone a 500-kb inverted duplication. The duplicated *NAIP* copy appears to be a pseudogene [17]. This duplication is specific to humans, while in other primates a pericentromeric inversion (chimpanzee and bonobo) and a translocation to Chromosome 19 (gorilla) have repositioned *NAIP* [18]. In mouse, *Naip* has expanded in gene number with five tandem copies reported in the C57BL/6J (B6) array *(mNaipa/b/c/e/f),* and at least seven in the 129 strain [19]. Gene order and orientation surrounding the *NAIP* locus in primates and rodents is preserved. Combined, these data suggest that *NAIP* is encoded within a region undergoing rapid evolution and is a good model to study both evolutionary processes and disease.

Deregulation of genes controlling apoptosis can lead to detrimental outcomes. *NAIP,* in particular, has been implicated in several diseases. Originally cloned in search of the spinal muscular atrophy gene [17], *NAIP* is now known to be a modifier of disease severity [13]. Also, a *NAIP* copy governs permissiveness of Legionella pneumophila replication in mouse macrophages, leading to Legionnaire's disease [20]. Finally, a role for IAPs as diagnostic and therapeutic cancer targets is emerging [13].

Here, we have studied transcriptional regulation of rodent and human *NAIP* genes and showed that LTR elements have repeatedly targeted and been coopted as promoters for these genes. Remarkably, these LTRs have been independently acquired during primate and rodent evolution. We also show that the 5′ flanking regions of all IAP genes are enriched for LTR-derived sequence compared to all genes. To account for these findings, we offer several possible scenarios, including the suggestion that utilization of LTR promoters by *NAIP* may be evolutionarily favored due to this gene's anti-apoptotic function.

## Results

### Transcription of Mammalian *NAIP* Genes Initiates within LTRs

**Figure 1:**
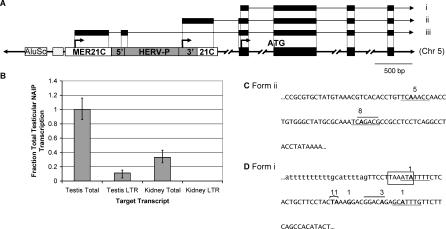
Contribution of LTR Promoters to Human *NAIP* Transcription and a Summary of 5′ RACE Results (A) Representation of a 5′ region of human *NAIP* gene. Transcription initiates at arrows situated above the underlying genomic DNA, with representative RNAs pictured above. Black boxes represent exons in DNA and RNA forms. White boxes represent a solitary MER21C LTR into which a HERV-P element has inserted (gray box). Sections of the HERV-P labeled 5′ and 3′ represent the 5′ and 3′ LTRs of this partly deleted ERV. Both the MER21C and the HERV-P are oriented in the same transcriptional direction as the *NAIP* gene. The boxes to the left of the MER21C denote an AluSc SINE and an MIR SINE (unlabeled). Three TSSs for human *NAIP* have been reported or were identified here: isoform i is found in all tissues tested, while ii represents the testis-specific HERV-P start site, and iii represents the published TSS determined in the THP1 leukemic cell line [21]. (B) Quantitative real-time RT-PCR analysis of human testis and kidney cDNA to determine contribution of the HERV-P LTR promoter to total *NAIP* transcription. Total transcript levels were determined using primers that amplify all of the most prevalent transcript forms, and LTR-driven transcripts were determined using one primer in the LTR (see Figure S1A for locations of primers and Materials and Methods for details). Expression levels are normalized to *GAPDH* and represented relative to total *NAIP* transcript levels in testis. Assays were carried out in duplicate and repeated three times in testis and two times in kidney. (C) Partial sequence of the HERV-P element (5′ end corresponds to Chromosome 5: 70,355,179 of the human March 2006 genomic assembly) underlying testis-specific TSSs of *NAIP.* The numbers of sequenced 5′ RACE clones aligning to particular TSSs are shown above the sequence. The putative TATA box identified previously in HERV-P LTRs [26] is at the end of the sequence shown. (D) Underlying sequence and TSSs determined for the non-LTR promoter (Chromosome 5: 70,352,387) in blood, liver, placenta, and testis. Lowercase letters distinguish intron/exon boundary. Two 5′ RACE clones aligned upstream of the intronic sequence shown. Numbers above boldfaced nucleotides indicate sites of transcription and the number of 5′ RACE clones that align to each TSS. Underlines and overlines indicate putative initiator elements and downstream promoter elements, respectively [25]. Boxed sequence represents a putative TATA box. Full characterization of human UTRs can be found in Figure S1.

In a screen of mouse and human gene expression databases similar to a previous study [6], we identified *NAIP* as an example of a gene with transcripts initiating within LTR sequence, suggesting potential use of the LTR as a promoter. Surprisingly, EST and RefSeq data suggested that human and mouse had recruited completely unrelated LTRs as promoters for these orthologs. The published human *NAIP* transcription start site (TSS) reported by Xu et al. [21] overlies a MER21C solitary LTR, which is itself interrupted by a HUERS-P3/LTR-9 element as annotated by Repbase (see Figure 1A) [22]. This latter ERV family has previously been termed HERV-P [23], and we will use this nomenclature throughout. While transcriptional regulation of the mouse *Naip* genes has not been studied in great detail, database transcripts initiate from a solitary ORR1E LTR of the MaLR superfamily [24]. The potential usage of different LTRs in regulation of mammalian gene orthologs has not been documented previously, and this fact prompted a further investigation to confirm and extend our bioinformatics screens.

Although the human *NAIP* TSS and promoter active in the THP1 leukemic cell line had previously been characterized [21] (Figure 1A, form iii), the LTR nature of the underlying sequence had escaped notice. We screened primary RNA samples from human blood, colon, placenta, and testis by 5′ rapid amplification of cDNA ends (RACE) and could not confirm this TSS in these tissues. As Xu et al. did, we also attempted to localize a 5′ start site in the region by RT-PCR using successively tiled primers along the length of the MER21C/HERV-P, and extending beyond its 5′ flank, combined with a common reverse primer. This analysis of blood, placenta, and testis cDNA yielded numerous products, due to the repetitive nature of the target sequence. Using Southern blotting, we resolved specific products for all primer sets across the MER21C/HERV-P complex. In addition, one primer upstream of this complex, located between the adjacent MIR short interspersed element (SINE) and nearby AluSc SINE, also gave a product of the expected size, but a primer upstream of the AluSc did not (see Figure 1A and unpublished data). These data suggest that a *NAIP* promoter may exist which incorporates SINE and LTR sequences into a repeat-rich 5′ UTR. While 5′ RACE was unsuccessful in confirming the previously published start site (Figure 1A, form iii), evidence for at least two other promoters was discovered. The principal TSS in all tissues tested (Figure 1A, form i), also strongly supported by EST data, lies within the third exon of the published cDNA from THP1 cells (Figure 1A, form iii), suggesting that the major promoter is upstream of this TSS. In testis, we identified two other closely spaced TSSs: remarkably they lie within the same MER21C/HERV-P complex but are located in the 3′ LTR of the HERV-P element, suggesting use of this LTR as an alternative promoter (Figure 1A, form ii)**.** One of these HERV-P TSSs is supported by a testis EST. Using quantitative RT-PCR, we determined that this HERV-P LTR promoter is responsible for ∼12% of total *NAIP* transcripts in normal testis but none were detectable in kidney (Figure 1B). We also confirmed by RT-PCR that a full-length transcript encoding an intact NAIP open reading frame (ORF) is produced from the LTR promoter (unpublished data). Various transcriptional regulatory features such as a putative TATA box and initiator and downstream promoter elements [25] were identified in the sequence underlying sites of LTR and non-LTR *NAIP* transcription (Figure 1C and 1D). Interestingly, the 5′ most TSS within the HERV-P LTR overlies an initiator element [25] that overlaps the putative CCAAT box previously detected in other members of this family of LTRs [26]. However, the putative TATA box identified in that study, while present in our example, does not appear to be used, as it is located downstream of the TSSs identified by 5′ RACE. These features and the extents of all 5′ RACE clones are shown in Figure 1C. To verify our identified *NAIP* TSSs, we checked the “cap analysis of gene expression” (CAGE) database [27] for mapped TSSs for the human *NAIP* gene, but none were found.

**Figure 2:**
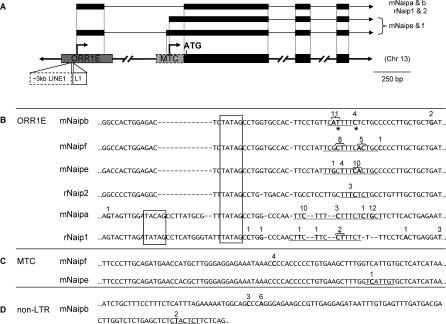
Contribution of LTR Promoters to Mouse and Rat *Naip* Transcription and a Summary of 5′ RACE Results (A) Representation of 5′ region of rodent *Naip* genes. Transcription initiates at arrows situated above the underlying genomic DNA, with representative RNAs pictured above. Gray shaded boxes represent the solitary LTR insertions, and black boxes represent exons in DNA and RNA forms. Mouse and rat *Naip* transcription predominately initiates in ORR1E LTRs. *mNaipe* and *mNaipf* have an MTC LTR (dashed gray box) and ∼3 kb of L1_Mus1 LINE1 sequence has integrated into the ORR1E LTRs associated with these two genes, shown by a dashed white box. The *rNaip2* ORR1E LTR has also been interrupted by an independent insertion of 300 bp of Lx2A1 LINE1, shown by solid white box. (B) Partial alignment of the rodent ORR1E LTRs associated with *Naip* transcription. The 5′ end of the sequences shown corresponds to the following coordinates in the mouse (mm8) and rat (rn4) draft sequences. (*mNaipa* = Chromosome 13: 101,553,198; *mNaipb* = Chromosome 13: 101,302,420; *mNaipe* = Chromosome 13: 101,347,641; *mNaipf* = Chromosome 13: 101,418,005; *rNaip1* = Chromosome 2: 31,268,656; *rNaip2* = Chromosome 2: 31,204,793). Numbers above boldfaced nucleotides indicate sites of transcription initiation and the number of 5′ RACE clones obtained that align to each TSS. A few *mNaipe* clones aligned beyond the boundaries of the ORR1E sequence shown. Underlines indicate putative initiator elements and boxed sequence represents putative TATA boxes. Asterisks denote sites of transcription that are supported by >1 CAGE tag [27]. (C) Partial alignment of the *mNaipe/f* MTC alternative promoters. (*mNaipe* = Chromosome 13: 101,346,591; *mNaipf* = Chromosome 13: 101,416,943). (D) Genomic sequence surrounding the *mNaipb* non-LTR promoter (*mNaipb* = Chromosome 13: 101,289,682). Full characterization of mouse UTRs can be found in Figure S2.

As mentioned above, database screens suggested that transcription of the mouse *Naip* genes initiates within an ORR1E LTR common to all mouse copies. We conducted 5′ RACE on primary B6 colon and liver RNA using primers specific for each *NAIP* copy *(mNaipa/b/c/e/f)* [19]. No evidence of *mNaipc* transcription was detected and it may represent a pseudogene detected through genomic Southern blots [19]. For all other mouse *Naip* genes, the major TSSs mapped within the common ORR1E LTR, confirming the database screens (Figure 2A). Due to the conserved position of a motif resembling a TATA box, sequence identity of flanking nucleotides, and localization of most TSSs 25–32 bp downstream of the TATA motif for all *mNaip* copies, these LTRs appear to be typical TATA box promoters (Figure 2B). The *mNaipb* gene is the only mouse gene with more than one CAGE tag, and two clusters of these tags correspond very well to our identified TSSs (Figure 2B). This 5′ RACE analysis also uncovered two alternative promoters for some of the mouse genes, one of which is a second LTR. The progenitor of the *mNaip*e/*f* paralogs was targeted by an MTC LTR [24], immediately 5′ of the first coding exon, prior to the duplication that created these two genes (Figure 2A and 2C). We found unique TSSs for each of these genes mapping within this LTR, suggesting its use as an alternative promoter. Finally, a minority of *mNaipb* transcripts initiate from a non-LTR promoter downstream of the initiation codon (Figure 2D), but within the first coding exon. The putative novel protein deriving from this isoform (not shown in Figure 2A) could potentially utilize a downstream initiation codon, resulting in an N-terminal truncated peptide encoding only the third BIR domain followed by the NBS and LRR motifs. Positions of 5′ RACE clones, as well as surrounding transcriptional regulatory features, are summarized in Figure 2B–2D. Unfortunately, MaLR LTRs have not been characterized for their regulatory signals; therefore, we could not compare our results to other functional studies.

Very little is known about *NAIP* transcription in rat and only a partial cDNA has been deposited in the database [28]. However, ECGene gene prediction software (University of California Santa Cruz Genome Browser) suggests that two tandem copies exist, which we have termed *rNaip1* and *rNaip2.* Based on these predictions, reverse primers were designed and 5′ RACE was carried out on rat spleen RNA. This analysis confirmed expression of both rat genes in the spleen and found that each initiates within an ORR1E LTR, analogous to the mouse genes (Figure 2A). Figure 2B aligns the mouse and rat ORR1E LTR regions encompassing the 5′ termini of all RACE clones and shows putative regulatory features.

### Tissue Distribution of *NAIP* Expression

**Figure 3:**
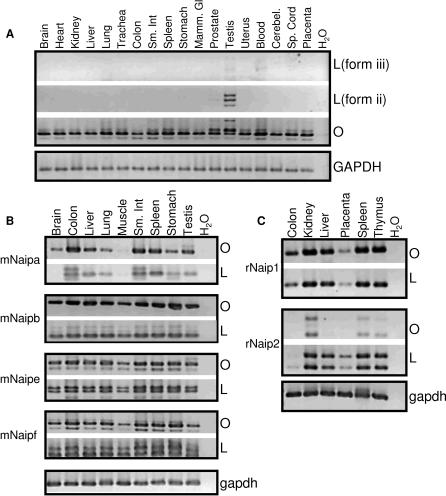
Transcriptional Profile of Human (A), Mouse (B), and Rat (C) *NAIP* across the Indicated Primary Tissues Primers selective for LTR-derived transcripts (L) or coding sequence (O) determined the breadth of LTR promoter use in all tissues in all organisms. In (A), L(form iii) primers were specific for the MER21C LTR-transcribed form and L(form ii) primers were specific for the HERV-P form. A *GAPDH* control is shown at the bottom of each panel.

To better understand the breadth of use of the human, mouse, and rat LTR promoters, we screened a broad panel of tissues by RT-PCR. Two sets of primers were used: one set selectively amplified LTR-derived transcripts, and the other set spanned protein coding exons to measure total gene expression (including transcripts deriving from alternative promoters). In human, constitutive expression of the *NAIP* coding region was observed in all tissues screened (Figure 3A, panel O). Using primers specific for the HERV-P-initiated form (Figure 1A, form ii), we detected transcripts in testis, as expected, and a low level in prostate, but in no other tissues, as shown in Figure 3A, form L(ii). Interestingly, the HERV-P family in general has been shown to be expressed in testis, prostate, and brain [23]. Using primers specific for the transcripts previously characterized by Roy et al. [17] and Xu et al. [21] (Figure 1A, form iii), we found only very faint signals in blood, lung, and testis as shown in Figure 3A, form L(iii). Due to the requirement of one primer annealing to repetitive DNA, we verified identity of all PCR products by sequencing. Several alternative exons deriving from repetitive DNA were discovered in both the UTR and ORF and are summarized in Figure S1.

Similar RT-PCRs were also performed for the individual mouse and rat gene copies across numerous tissues. Due to the very high overall sequence identity of these genes, the specificity of RT-PCR products was confirmed by sequencing. In all cases, the pattern of expression for the ORR1E-driven transcript forms was very similar to the pattern obtained using primers within coding regions, suggesting that the ORR1E LTRs are the major *Naip* promoters (Figure 3B and 3C). We verified the *mNaip* TSSs by RT-PCR with primers upstream of the putative TATA boxes, and, as expected, observed no RT-PCR products (unpublished data). A panel including more mouse tissues, with respect to the one shown (Figure 3B), also showed a very similar pattern of expression using the different primer sets (unpublished data). Various splice isoforms identified among the mouse copies also incorporate exons deriving from both repetitive and nonrepetitive DNA, summarized in Figure S2.

### Promoter Activity of the ORR1E LTRs

**Figure 4:**
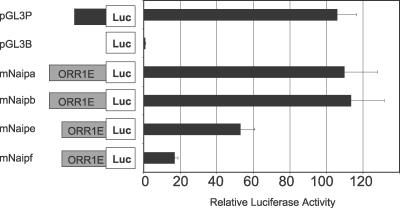
Promoter Activity of the *mNaip* LTRs The ORR1E LTRs for each copy were cloned into a modified pGL3B vector and tested for luciferase activity in the MS1 cell line. pGL3B and pGL3P, containing a SV40 promoter, were used as negative and positive controls, respectively. Luciferase activity was normalized relative to the cotransfected *Renilla* luciferase control and then to pGL3B to demonstrate fold activation. Each bar represents the mean of at least four independent transfections ± SEM.

In other reported cases of LTRs acting as promoters for cellular genes, the LTR has been a minor or tissue-specific promoter [7,8]. The fact that the rodent *Naip* genes appear to employ an LTR as a primary constitutive promoter is therefore highly unusual. To confirm that mouse ORR1E LTRs possess promoter activity, reporter gene assays were performed. Constructs of the ORR1E LTRs for each mouse copy were tested in MS1, EL4, and RMA-E3 B6 cell lines. Although the scale of luciferase activity varied between cell lines, the same general trends were observed (Figure 4, unpublished data). All tested constructs showed marked increases over a promoterless control, and the *mNaipa* and *mNaipb* LTR constructs were comparable in activity to the SV40 promoter. The *mNaipe* and *mNaipf* ORR1E LTR constructs had lower promoter activity but were also 5′ truncated by ∼100 bp because we did not include any of the intervening long interspersed element 1 (LINE1) sequences disrupting these ORRIE copies in our constructs (see Figure 2A). The fact that these truncated constructs have lower promoter activity could indicate the presence of positive regulatory element(s) within the 5′ terminus of these ORR1E LTRs, consistent with typical retroviral LTRs [29]. Subtle sequence differences also play a role in the different promoter activities since the highly similar *mNaipe* and *mNaipf* LTRs (97% identical) differ in promoter activity (Figure 4).

### Rapid Evolution of the *NAIP* Promoter Regions

**Figure 5:**
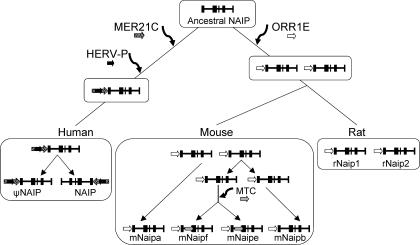
Association of LTR Elements with *NAIP* through Mammalian Evolution A single *NAIP* progenitor was present in the last common ancestor of primates and rodents. Following the primate/rodent split, *NAIP* was independently targeted by multiple lineage-specific LTRs. In human, *NAIP* is part of a large inverted duplication but the centromeric copy is a pseudogene. In rodents, this locus duplicated prior to mouse-rat divergence. In mouse, *Naip* has undergone further expansion, where the two youngest copies, *mNaipe* and *f,* acquired the MTC LTR.

A likely evolutionary scheme to explain association of the LTR elements with mammalian *NAIP* genes is shown in Figure 5. The MER21C and HERV-P elements must have inserted upstream of the ancestral primate *NAIP* gene at least 40 million y ago since both are present in Old World (human, chimpanzee, Rhesus monkey) and New World (marmoset) primates, according to genome database comparisons (unpublished data). The most probable scenario to explain the presence of ORR1E LTRs upstream of all rodent *Naip* genes is that the element inserted upstream of the ancestral rodent gene and then was included in subsequent duplication events involving the gene. At a later stage, the *mNaipe/f* progenitor acquired an MTC LTR (Figure 5). Interestingly, alignments of the four mouse and two rat ORR1E LTRs reveal that *mNaipb/e/f* and *rNaip2* are ∼85% identical to each other, and a similar level of identity exists between *mNaipa* and *rNaip1.* In contrast, *mNaipb/e/f:mNaipa* and *rNaip2:rNaip1* LTR copies are less similar to each other, exhibiting 60%–65% identity, an unusual finding considering that a similar sized repeat-free noncoding segment of intron 8 from *rNaip1/2* and *mNaipa/b* exhibits nucleotide identity on the order of 90% among all copies. Moreover, comparisons of the various rodent *Naip* gene-coding regions *(rNaip 1* and *2* and *mNaipa* and *b)* also give levels of nucleotide identity of ∼90% (unpublished data) and do not clearly distinguish orthologous gene pairs. These data suggest that gene conversion events have homogenized the genomic sequence encoding *Naip,* obscuring the evolutionary relationships of intronic and coding regions. While we assume that the ORR1E LTRs associated with these genes derive from a single ancestral insertion, we also addressed, by phylogenetics, the less likely possibility that the present LTR-gene arrangements arose by independent insertion of different ORR1E LTRs into progenitors of *mNaipa/rNaip1* and *mNaipb/rNaip2.* Unfortunately, the age and divergence of these and other MaLRs, coupled with extensive genomic rearrangements in the region, hindered phylogenetic analyses and comparisons of flanking regions. However, the rodent *Naip* ORR1Es are more similar to one another, than to others present in either genome, supporting the premise that they derive from one original insertion.

**Figure 6:**
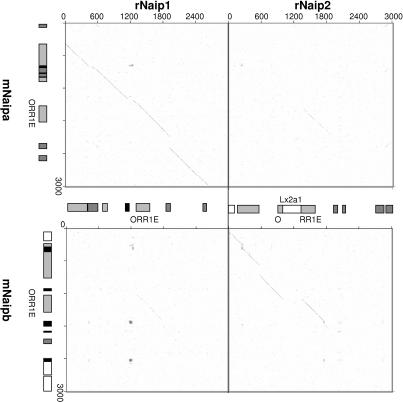
Comparison of Genomic Sequence Surrounding the Rodent *Naip* ORR1E LTRs 3 kb of sequence centered around the ORR1Es was analyzed by dot plots; diagonal lines represent regions of homology between compared sequences. Light gray, dark gray, white, and black boxes represent LTR elements, SINEs, LINEs, and simple repeats, respectively.

While segments of the ORR1E elements have been retained, their genomic environments have been subjected to repeated disruption by rearrangements and other TE insertions. This is illustrated in Figure 6, in which DNA sequences surrounding each LTR are compared using dot plots. This analysis demonstrates that the 5′ regions flanking *mNaipa:rNaip1* and *mNaipb:rNaip2* are orthologous, as the lines of homology are more robust than between reciprocal dot plots. This agrees with the sequence comparisons of the individual LTRs. All combinations of dot plots comparing sequence surrounding the ORR1E LTRs of rodent *Naip* paralogs revealed a line of homology beginning near the annotated start of the LTRs and extending to a common point ∼150 bp beyond the annotated ends, with no other significant similarity in the regions. It would seem that only parts of the LTR and the flanking ∼150-bp region have been retained amid rapid turnover of surrounding sequences.

Dot plots across the entirety of genomic DNA encoding the rodent *Naip* genes revealed that most of the retrotransposon integrations are not shared among orthologs/paralogs. In fact, only for orthologs such as *mNaipa* and *rNaip1* is the TE repertoire mostly in common (Figure 6 and unpublished data); all other copies bear little resemblance. One interesting feature is the fact that the *mNaipe/f* and *rNaip2* ORR1E LTR promoters have retained different LINEs at near corresponding positions, upstream of the TSSs (see Figure 2A). It is not known if these LINEs have any effect on the promoter function of the LTRs.

### Retroelement Prevalence in IAP Gene 5′ Flanking Regions

**Figure 7:**
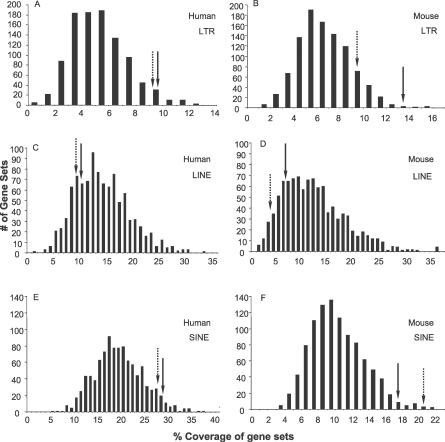
Density of TE Sequence in 5′ Flanking Regions of IAP Genes Compared to Random Gene Sets Coverage of LTRs, LINEs, and SINEs in human (A, C, and E) and mouse (B, D, and F) was assessed in a 12.5-kb window surrounding database-annotated TSSs, 10 kb upstream and 2.5 kb downstream of the eight human and eight mouse IAP genes. These values, shown by solid arrows, were compared to the coverage of each type of repeat for 1,000 sets of eight random human and eight random mouse genes. For the human IAP genes, while SINE enrichment approaches significance (95th percentile), LTRs are significantly enriched (97th percentile), and LINEs are not overrepresented (20th percentile) within the analyzed windows. For the mouse IAP genes, both LTRs (99th percentile) and SINEs (98th percentile) are significantly enriched around the IAP 5′ termini, while LINEs are not (18th percentile). Dashed arrows show retroelement coverage in the same window for IAP genes when the *NAIP* genes themselves are removed from the analysis.

The fact that human and rodent *NAIP* genes have independently coopted different LTRs as promoters is extremely unusual, and prompted us to ask whether the anti-apoptotic function of these genes could somehow have increased the probability of such cooption events. For example, if such genes are generally enriched for LTR elements in their 5′ flanking genomic regions compared to genes at large, the probability that LTRs would be adopted as promoters would likely increase. We therefore computed the prevalence of LTRs and other retroelements in a 12.5-kb window of genomic sequence surrounding annotated TSSs of the eight human IAP family genes [13,14]: *NAIP (BIRC1)* and *BIRC2–8.* To put this result in context, we computed the distribution of LTR coverages for 1,000 sets of eight genes chosen at random (see Materials and Methods). The same analysis was performed for eight mouse IAP genes, a set including *mNaipa, mNaipb,* and *Birc2–7.* Importantly, we did not observe shared LTRs or other TE insertions between the different IAP family members, indicating the TE insertions were acquired independently (Table S1 and unpublished data). Figure 7A and 7B shows the distributions of total LTR-derived sequence coverage for the sets of randomly chosen genes, and LTR coverage for the IAP genes is indicated with an arrow. The upstream 12.5-kb regions of human IAP genes are significantly enriched in LTR sequence, which comprises 9.75% of the bases. This level of LTR coverage puts IAP genes in the 97th percentile compared to random gene sets (Figure 7A). For the mouse IAP gene set, LTR sequence covered 13.8% of the bases. Only three of the 1,000 random gene sets were higher in LTR coverage than this value (Figure 7B).

In addition to LTR elements, we performed an identical analysis for other types of retroelements. In contrast to LTRs, LINEs showed no enrichment in either human or mouse in IAP upstream regions (Figure 7C and 7D). Similar to LTRs, however, SINEs were overrepresented in the upstream regions of human and mouse IAP genes compared to random genes (Figure 7E and 7F).

We noted that *mNaipa* and *mNaipb* are particularly LTR-rich compared to the other IAP genes (Table S1). Therefore, to determine if retroelement coverage for the *NAIP* genes in particular was unusual, we repeated the same analyses, excluding the *NAIP* genes from the IAP gene groups. For the human IAP gene group, the fractional coverage by each retroelement type changed little when *NAIP* was excluded from consideration (Figure 7A, 7C, and 7E). By contrast, the high LTR coverage in the upstream region of mouse IAP genes ceases to be significant, upon exclusion of the mouse *NAIP* genes themselves from the IAP gene set, although it remains above the mean (83rd percentile; Figure 7B).

## Discussion

Here, we have demonstrated that different endogenous LTRs serve as promoters of the mammalian *NAIP* genes. A recent study utilizing a large dataset of human and mouse TSSs generated by the CAGE approach [27] has found that TSSs are subject to rapid evolutionary turnover and that some orthologous genes have TSSs in completely different positions [30]. The *NAIP* genes are an example of such genes. It is also worth noting that the CAGE approach might miss some start sites provided by LTRs or other TEs due to difficulty in uniquely mapping short tags containing repetitive sequence, unless such TEs are sufficiently diverged from other copies. Indeed, the fact that the ORR1E LTR TSSs for the *mNaipb* gene are supported by CAGE tags (Figure 2B), is due to the fact that this LTR is diverged from other copies in the genome. Thus, it is possible that a significant number of TE-derived TSSs remain to be detected.

For the few mammalian genes where use of an LTR as a promoter has been demonstrated, two typical situations exist. In the first scenario, an ancient LTR present in both human and mouse serves as a promoter for the orthologous genes. An example is the carbonic anhydrase gene *(CA1),* where an ancestral LTR drives erythroid-specific expression of the orthologs [6]. The more commonly documented situation is where a lineage-specific LTR acts as a gene's promoter in one species but not the other, as illustrated by the *β3GALT5* gene in human [31] and various mouse genes including *Spindlin* [32]. The results of this study illustrate a third evolutionary scenario not previously reported: distinct LTR elements specific to the primate or rodent lineages have independently assumed roles as promoters for the *NAIP* orthologs.

In human, *NAIP* was originally cloned from a fetal brain cDNA library [17], and the 5′ and 3′ termini were subsequently resolved [33]. We noticed that the 5′ terminus of this form and the 432-bp 5′ extended form identified by Xu et al. [21] in the THP1 leukemic cell line, localized within a MER21C LTR. While unable to confirm these TSSs, we did observe a variant *NAIP* transcript which includes and extends upstream of the MER21C and adjacent MIR SINE (unpublished data). This may simply be a result of spurious transcription, reportedly commonplace throughout the human genome [34]. Alternatively, it could point to existence of yet another *NAIP* promoter that could not be identified by 5′ RACE due to a size constraint or complex secondary structure. Surprisingly, through 5′ RACE we discovered that the HERV-P 3′ LTR imbedded within the MER21C element appears to be a functional promoter in testis. Earlier work identified *NAIP* expression in liver and placenta by Northern blot using a coding region probe and in spinal cord and lymphoblasts following nested RT-PCR spanning coding exons [17]. Our expression screens by RT-PCR of a broad panel of tissues confirmed these findings and extended them to include all tested tissues. Constitutive *NAIP* expression most likely initiates within the non-LTR promoter we have identified here. Quantitative RT-PCR indicated that, in normal testis, the HERV-P LTR is a significant but relatively minor *NAIP* promoter (Figure 1B). Nonetheless, the activity of this LTR promoter in testis, and previous description of the MER21C LTR promoter active in a leukemic cell line [21], coupled with reports of elevated *NAIP* expression in myelodysplastic syndromes and leukemia [35,36], provides an enticing model to study potential upregulation of these LTR promoters in certain forms of cancer, possibly through hypomethylation, since both LTRs are CpG-rich (unpublished data).

In rodents, the results presented here demonstrate that the mouse and rat *Naip* genes employ a common ORR1E LTR as their major promoter. ORR1s and MTs are rodent-specific LTR families within the MaLR superfamily [24], represented by >400,000 copies in the sequenced mouse genome [2]. The fact that the ORR1E LTR is the primary promoter for these genes is unusual, considering LTRs most often function as tissue-specific or alternative promoters [7,8,31,32,37]. Another intriguing finding is the fact that an MTC LTR has inserted into the *mNaipe/f* progenitor and behaves as a secondary promoter. Thus, the *NAIP* locus represents an extremely rare case of repeated recruitment of distinct LTRs as promoters during the course of mammalian evolution.

In a previous study, we found that more rapidly evolving genes or mammalian-specific genes are more likely to incorporate TEs into their UTRs, compared to genes at large [6]. *NAIP* represents an example of such a gene since no nonmammalian ortholog is known and its rate of protein evolution as measured by a human-rodent Ka/Ks value of 0.44 (TAED Adaptive Evolution Database [38]) is above the median for all genes of 0.115 [2]. Ka/Ks is the normalized ratio of nonsynonymous to synonymous nucleotide substitution rates in coding sequence [2,38]). Nonetheless, assuming roughly 20,000 orthologous genes between humans and mice and a ∼0.7% frequency of human RefSeq genes employing LTR elements as promoters [6] (unpublished data), we predict just a single example of orthologous gene pairs having adopted lineage-specific LTR promoters by chance. Examples of the same primate locus acquiring independent Alu insertions have been reported [39], but we are unaware of other cases where distinct TEs provide regulatory function to orthologous genes. Remarkably, in both lineages, more than one LTR insertion contributes to *NAIP* promoter activity, a combination of events extremely unlikely to be due to chance alone. Several potential factors that could have contributed to this phenomenon are presented below.

The first factor could be that the region upstream of this gene is subject to a lower selective constraint compared to most other genes, resulting in TE accumulation and increasing the probability that some may assume a regulatory role. Indeed, the fact that *NAIP* is part of the IAP gene family, with potentially overlapping or redundant functions, may have resulted in increased host tolerance to regulatory change of any individual family member. Supporting this possibility is the fact that genomic coverage by LTR sequences and SINEs upstream of human and mouse IAP genes is above average (Figure 7). Moreover, the tandemly duplicated mouse *Naip* genes have a higher LTR coverage and insertion number relative to most other mouse IAP genes (Table S1). These genes represent the high end of the genomic spectrum in terms of LTR and SINE density, which could indicate that their regulatory requirements are flexible and localized to small domains. Representing the opposite end of the spectrum are *Hox* genes and other critical transcription factor genes or developmental genes which are located in regions nearly devoid of all TEs [1,40], likely because their complex regulation requires extended regions to be free of interruptions.

Interestingly, while LTR and SINE density 5′ of IAP genes is above average, LINE density is not (Figure 7), indicating that not all TEs have accumulated in the region. In addition, the high density of SINEs upstream of IAP genes may be related to the known role of the highly repetitive SINE sequences in facilitating genomic rearrangements [41]. The BIR domain was amplified to create the IAP family, *NAIP* genes have amplified variably in rodents [19], and two other IAPs, *cIAP1* and *2,* are tandemly duplicated copies present in primates and rodents [42], implicating ongoing genomic rearrangements in IAP gene expansion. Moreover, while the IAP genes are classified as a gene family due to the shared BIR domain, mouse gene knockout evidence suggests these proteins do not encode entirely overlapping functions. When only *mNaipa* is deleted, mice display poor neuronal survival under pathological conditions [43]. However, the effect of eliminating all *mNaip* copies remains unknown. Deletion of two other IAP family members, *Survivin* [44] and *Bruce* [45] result in embryonic lethality. *XIAP-*deficient mice develop normally [46], but recent reports indicate that it encodes a nonredundant function related to TRAIL-mediated apoptotic signaling [47]. Targeting of the *cIAP2* locus leads to a defective innate immune response [48]. Finally*, ML-IAP* is overexpressed in human melanoma cells [49] and *Ts-IAP* expression is testis-specific [50]. These nonoverlapping phenotypes indicate that some degree of selection must operate on their regulatory regions.

A second potential explanation is that, compared to most genes, the 5′ flanking regions of *NAIP* may have been more receptive to initial retroviral or retroelement insertion, increasing the chance of LTR recruitment by this gene. Different classes of retroviruses and retroelements have distinct integration site preferences [51]. For example, HIV favors integration within active genes, murine leukemia virus favors the 5′ ends of genes, Ty1 and Ty3 LTR retroelements of Saccharomyces cerevisiae target regions upstream of pol III-transcribed genes, and Ty5 targets heterochromatic regions [51]. An interesting recent report has documented that promoters of heat-shock genes in *Drosophila* are particularly prone to insertions by P elements, a very young family of DNA transposons, likely at least in part due to the unusual constitutively open chromatin associated with these genes [52]. In the case under study here, since the HERV-P and MER21C elements upstream of the primate *NAIP* gene are members of the broad “class I” subdivision of ERVs [22], which also include murine leukemia virus, it is possible that these ERVs also prefer 5′ flanks of genes for integration. On the other hand, the rodent ORR1E and MTC LTRs of the MaLR superfamily (class III in Repbase nomenclature [22]) are not related to any elements with known integration site preferences, thus we cannot speculate as to whether such elements may have originally favored regions upstream of genes. It is known that the overall genomic densities of class III elements are highest in regions further from genes compared to other ERV classes [53]. Furthermore, it seems unlikely that the upstream region of *NAIP* specifically, compared to all genes, would present a favored integration target for widely different retroviral types in different species.

Since it is generally assumed that the genomic distribution patterns of ancient ERVs are shaped by selection and bear little resemblance to their original integration site preferences that are unknown, a third hypothesis to account for repeated LTR cooption by *NAIP* is based on this gene's function. Perhaps utilization of retroviral LTRs as promoters for *NAIP* is somehow advantageous to the host, resulting in their selective retention during evolution. For example, activation of *NAIP* via an LTR promoter may provide an avenue for germ cells to escape transitory, stress-induced apoptotic signals. LTR promoters may be particularly responsive to upregulation by cellular stresses since it has been shown that activation of human and mouse ERV LTRs can occur following stresses such as viral infection [54–56] and UV irradiation [57,58]. Various IAPs are expressed in human [59,60], mouse [61], and rat [62,63] germ cells or their progenitors, and it has been reported that *Naip* expression plays a role in mouse oocyte viability [61]. Although nothing is known about a potential NAIP stress response in the germ line, it has been demonstrated that *NAIP* mRNA and protein is upregulated in neurons following ischemic stress [64]. It is also interesting that activity of the human *NAIP* HERV-P LTR promoter is highest in testis, and, in general, ERVs are transcribed highly in germ cells and early embryogenesis compared to most normal somatic cells [32,65]. While there is no evidence that other IAP genes, with the exception of *NAIP,* use LTR promoters, the proposed upregulation may involve gene activation by nearby LTR enhancers, offering an explanation for the fact that LTR density upstream of IAP genes as a group is high compared to random genes. Alternatively, *NAIP* may be unique among IAP genes in retaining LTR promoters because of its specialized functions or flexibility in regulatory control.

Finally, a related, but much more speculative hypothesis to explain LTR usage by the *NAIP* genes postulates that the present state reflects a viral mechanism to evade apoptosis. Infection by retroviruses can lead to induction of apoptosis [66,67], and HIV Nef activates caspases [66], the targets of IAP proteins. Waves of intracellular retrotransposition can also be associated with increased apoptosis [68]. Therefore, retroviral/retroelement insertions in germ line cells which, by chance, induce expression of anti-apoptotic genes, could abort an initial or transitory stress-induced apoptotic response, increasing the probability that cells harboring such insertions would survive and contribute to subsequent generations, assuming they have not suffered damage. In such a scenario, an LTR would only need to exert regulatory effects for a short window in time immediately after insertion, before being silenced (for example, by DNA methylation), or it could continue to be used as a promoter if such activity is not detrimental to the organism, as in the case of the *NAIP* genes. Viruses have evolved numerous ways of circumventing host defense strategies and aborting apoptosis [69]. Indeed, one such example is the viral origin of the anti-apoptotic BIR domain, shared by all IAP genes [70]. Perhaps repeated targeting of LTR elements to regulatory regions of *NAIP* genes represents another viral mechanism aimed at maintaining cellular viability. Nonetheless, retroviral or other TE insertions in the germ line will not be tolerated by the host species unless they are neutral and fixed by random chance, or are advantageous. Thus, such hypothetical scenarios are tenable only if the LTR insertions do not have a detrimental impact on cell function or on organismal development.

In conclusion, we have shown here that ERV LTRs have been repeatedly coopted to serve regulatory roles for the mammalian *NAIP* genes and presented various potential explanations to account for this phenomenon. These results document a striking example of how ancient ERV insertions can be domesticated or “exapted” [9,10] by the host, contributing to gene regulatory evolution.

## Materials and Methods

### RNA isolation.

Primary mouse tissue samples were dissected from healthy adult male C57BL/6J (B6) mice, and preserved in RNA Later (Ambion, http://www.ambion.com). All samples were processed using TRIzol (Invitrogen, http://www.invitrogen.com), except peripheral blood leukocytes for which the QIAamp RNA Blood Mini Kit was used (Qiagen, http://www1.qiagen.com). B6 testis, Sprague Dawley rat, and all human RNA samples were purchased from Clontech (http://www.clontech.com), with the exception of primary human blood and placenta samples. These were obtained from Dr. C. Eaves (Terry Fox Laboratory) and Dr. P. Medstrand (Lund University, Sweden), respectively.

### 5′ RACE.

5′ RACE analysis of human blood, colon, placenta, and testis, B6 liver and placenta, and Sprague-Dawley spleen RNA was performed using the FirstChoice RLM-RACE kit (Ambion). Manufacturer's recommendations were followed, but on occasion several kit components (calf intestinal phosphatase [CIP], RNA ligase, and MuLV reverse transcriptase [RT]) were substituted for CIP (NEBiolabs, http://www.neb.com), RNA ligase (NEBiolabs), and SSIII RT (Invitrogen) laboratory stocks. Gene-specific reverse primers and reaction conditions are summarized in Table S2.

### Genomic PCR and generation of constructs.

Genomic DNA (gDNA) was isolated from B6 liver using DNAzol (Invitrogen) as outlined by the manufacturer. Only the ORR1E LTR of each *Naip* copy was amplified using Platinum Taq HIFI (Invitrogen) as outlined by the manufacturer. *mNaipe/f* LTR constructs were 5′ truncated by ∼100 bp due to ∼3 kb of intervening LINE1 sequence, which we opted not to include. PCR reaction conditions and primers used for amplification of fragments are listed in Table S2. Primers incorporated AflII and HindIII restriction enzyme recognition sequences to facilitate directional cloning into a modified pGL3B (Promega, http://www.promega.com/default.asp) vector. All constructs were sequenced to verify their fidelity. Our pGL3B promterless vector is a slight modification of the manufacturer's and has been published elsewhere [71]. Briefly, the multiple cloning site was replaced with a series of strong polyadenylation signals, to reduce background luciferase expression.

### Cell culture and luciferase assays.

All cell lines assayed were B6-derived: MS1 (pancreatic), EL4 and RMA-E3 (lymphoid). Cells were cultured in DMEM (StemCell Technologies, http://www.stemcell.com) supplemented with 10% fetal bovine serum (Invitrogen) and grown at 37 °C under 5% CO_2_. Cell stocks were maintained in penycillin/streptomycin, but all transfection experiments were carried out in its absence.

Prior to transfection, suspension cells (EL4 and RMA-E3) were seeded at 500,000 cells per well and adherent cells (MS1) at 50,000 cells, in 24 well plates. Lipofectamine (Invitrogen) and Lipofectamine 2000 (Invitrogen) targeted our constructs to adherent and suspension cells, respectively, according to manufacturer's guidelines. Approximately 24 h post-transfection, the cells were washed with PBS (StemCell Technologies), processed, and analyzed for firefly and *Renilla* expression using the Dual Luciferase Reporter Assay System (Promega). All values were standardized to the *Renilla* luciferase internal control to assess transfection efficiency, then to the modified promoterless pGL3B construct.

### cDNA synthesis and RT-PCR.

Initial experiments used SuperscriptII (Invitrogen) reverse transcribed RNA as described elsewhere [37]. These findings were confirmed by SuperscriptIII (Invitrogen) random hexamer-primed reverse transcribed RNA according to manufacturer's recommendations. cDNA amplification was carried out using Platinum Taq (Invitrogen) over 35 cycles. All primers and their associated annealing temperatures and extension times are summarized in Table S2.

### Quantitative RT-PCR.

The cDNA used for quantitative RT-PCR with Power SYBR Green PCR Master Mix (Applied Biosystems, http://www.appliedbiosystems.com) in the ABI 7500 Real Time PCR System (Applied Biosystems) was prepared as above. Stock primers were at a 10-μM concentration and they were determined to work equally efficiently, within a certain range of template dilution, using a standard curve. Consequently, the comparative C_T_ method was used for quantification of target (ORF and LTR-derived) versus a GAPDH endogenous control in testis and kidney. Each experiment was conducted three times, with at least two replicates per plate, and the cycling parameters were as follow: 50 °C, 2 min; 95 °C, 10 min; 95 °C, 15 s (40 cycles); 60 °C, 1 min. At the end of each run, dissociation curves were generated, which indicated the specificity of amplification, also verified by RT-PCR (unpublished data). Due to the difficulty of primer design posed by splicing variants (Figures 3A and S1), we were able to quantify only one of the HERV-P LTR-promoted forms (topmost band, Figure 3A and top form in Figure S1A), and estimated that it reflected half of the total LTR-derived transcripts. The value obtained was therefore doubled to deduce the total LTR-derived transcripts and this doubling is reflected in Figure 1B. Real-time primers are listed in Table S2, and they all begin with the prefix “q.”

### Sequencing.

PCR products and reporter constructs were cloned into the T-vector (Promega) or our modified pGL3B (Promega), respectively and sequenced at the McGill University sequencing facility. Sequencing verified that primers selectively amplified target genes and not their paralogs, with the exception of the lower band in *mNaipe* and *mNaipf* ORF RT-PCR panels (Figure 4B), identified as *mNaipb.* All sequences were stored and analyzed in the SDSC Biology Workbench (http://workbench.sdsc.edu), offering a suite of analytical tools.

### Dot plots.

DNA sequence surrounding the LTR promoters of mouse and rat *Naip (mNaip* and *rNaip)* were obtained from the UCSC Genome Browser (http://genome.ucsc.edu) using the February 2006 mouse genome assembly and the June 2003 rat genome assembly. Comparative analysis of genomic sequence was completed using the Web-based jdotter (http://athena.bioc.uvic.ca/workbench.php?tool=jdotter&db=). All dot plots were prepared using a 25-bp window and the greymap tool was iteratively adjusted to distinguish true lines of homology from background. Analyzed sequences were manually annotated across their lengths.

### Analysis of retroelements in 5′ flanking gene regions.

Overall base pair coverage by retroelements (LTRs, LINEs, and SINEs) in a 12.5-kb window (10 kb upstream and 2.5 kb downstream) surrounding the 5′ terminus of the longest annotated transcript of IAP family genes (delineated in EnsEMBL-v37) was determined. Annotation files generated by RepeatMasker (v3.1.4) from the May 2004 assembly of the human genome and the August 2005 assembly of the mouse genome were used to obtain pertinent attributes for all repeat elements. Base pair coverage by different retroelement classes among human and mouse IAP genes (eight in human, *BIRC1–8;* eight in mouse, *mNaipa/b* and *Birc2–7*) was compared to 1,000 randomly selected comparable-sized sets of genes. The *mNaipe/f* genes were excluded because they were recently duplicated from a *mNaipb*-like gene. Numbers of LTR insertions in the window for the human and mouse IAP genes (manually checked for accuracy) are shown in Table S1. However, because indels and rearrangements of ancient TEs hampered accurate automated tabulation of numbers of insertion events for the random sets of genes, we instead determined total base pair coverage by the three retroelement classes upstream of the IAP genes and random sets of genes. For ERV-like elements, we considered the LTR part only, because LTRs are known to harbor regulatory signals. We therefore excluded sequences annotated as ERV internal sequences, which are annotated in human with names including the text strings “ERVL,” “HERV,” “-int,” “Harlequin,” and “HUERS-.” In mouse, internal sequences were identified by names including the text strings “_I,” “-int,” and “ERV.”

## Supporting Information

Figure S1 Analysis of Human *NAIP* 5′ UTR and Coding Region Splice Isoforms Cloned RT-PCR products amplified by primers specific for the two alternative LTR-derived transcripts are shown.
(A) Represents RT-PCR products specific for the HERV-P-driven form (Figure 1A, form ii). The arrows show locations of primers used for quantitative real-time RT-PCR.
(B) Represents products from the MER21C-associated form (Figure 1A, form iii). Recruitment of a heterogeneous ERV (5′-HAL1/LINE:AluJb/SINE-3′) was detected in sequenced clones from these isoforms. We also observed occasional exclusion of the exon from which most 5′ RACE clones were found to initiate (Figure1A, form i). These UTR variants could not be compared to those reported by Xu et al. [21] as their sequences are not available.
(C) Splice variants identified by RT-PCR using primers specific for coding region exons are shown. Downstream of the first coding exon, 74 bp of a 102-bp remnant of an antisense MIRm SINE is recruited into the coding region of human *NAIP* in peripheral blood leukocytes. While verified by direct sequencing only in peripheral blood leukocytes, we infer transcription of this isoform in all tissues because the same band is seen in all lanes of our expression profiling experiment (Figure 4A, top band, panel O). This isoform does not preserve the established ORF (+292 to +4,503, relative to the transcript form previously reported [17,33]) and is predicted to yield a truncated protein encoding only the first and part of the second BIR domain (+292 to +888, relative to the previously reported transcript). However, downstream of the intervening MIRm SINE we report on a predicted ORF (+919 to +4,578) initiating at a start codon in-frame with the standard one (+292) that retains part of the second BIR, entire third BIR followed by the expected NBS and LRR motifs. Another minor isoform splices out the second coding exon, also disrupting the normal ORF, but utilizes an in-frame start codon to yield a novel predicted peptide (+993 to +4,412) encoding the third BIR and NBS and LRR motifs. In all diagrams, black boxes indicate nonrepeat-derived exons and colored boxes are repeat-derived exons with their identities labeled above. ATG denotes the accepted initiation codon for *NAIP.* AS, antisense.
(45 KB PPT)

Figure S2 Analysis of *mNaip* 5′ UTR and Coding Region Splice Isoforms (A) Cloned RT-PCR products amplified by primers specific for transcripts initiating within the ORR1E LTR are shown. Size of the ORR1E exon shows some variability among *mNaip* copies. Only *mNaipa/b* utilize a second, downstream exon within their 5′ UTRs (labeled 2). *mNaipb* also demonstrates recruitment of two other novel exons into its 5′ UTR, one of which utilizes partial B1F1/SINE sequence. Interestingly, we observe a *mNaipe* isoform that is not spliced across the length of its 5′ UTR; we are unable to comment whether it yields a functional protein, but might represent a primary transcript not yet processed by splicing machinery.
(B) Splice variants for each *mNaip* copy using primers across coding region exons are shown. All coordinates noted below are relative to the accession numbers of the mouse Naip transcripts listed in the Accession Number section. Similar to human, we find recruitment of a repetitive exon into the *mNaipa* coding region, here 129 bp of the 5′ segment of a 554-bp antisense Lx LINE remnant splices in downstream of the second coding exon. This novel exon introduces an in-frame stop codon and the resulting truncated protein (+113 to +904, relative to the reported *mNaipa* transcript) encodes only the first two BIR domains. In addition, a novel ORF (+1,023 to +4,442) where the new initiation codon downstream of the intervening Lx LINE is in-frame with the standard one (+113) could potentially be translated to encode a protein incorporating the third BIR domain followed by the NBS and LRR. Similarly truncated proteins are expected for the isoforms of *mNaipe* and *f* which splice out the second coding exon. The C-terminal truncated peptide (+200 to +847, relative to the reported *mNaipe* and *f* transcripts) terminates within the third coding exon and is predicted to encode the first and part of the second BIR. A start codon in-frame with the standard one (+200) within the fifth coding exon yields an ORF (+892 to +4,311) that encodes the third BIR, followed by the NBS and LRR. In all diagrams, black boxes indicate nonrepeat-derived exons and colored boxes are repeat-derived exons with their identities labeled above. ATG denotes the accepted initiation codon for *Naip.* AS, antisense.
(45 KB PPT)

Table S1 TRI Insertions within the Analyzed Windows for All Human and Mouse IAP Genes (29 KB DOC)

Table S2 Primers and Associated Information (25 KB XLS)

### Accession Numbers

Accession numbers used in this paper are from the National Center for Biotechnology Information (NCBI) (http://www.ncbi.nlm.nih.gov) database. Accession numbers (human/mouse) for the IAP genes used are: *bruce* or *BIRC6* (AF265555/Y17267); *cIAP1* or *BIRC2* (BX647978/U88909); *cIAP2* or *BIRC3* (AF070674/U88908); *livin* or *BIRC7* (AY358835/BC107260); *NAIP* or *BIRC1* (U19251/*mNaipa,* AF135491; *mNaipb,* AF135490; *mNaipe*, AF135492*;* and *mNaipf*, AF135494); *survivin* or *BIRC5* (CR612752/W97263); *TsIAP* or *BIRC8* (AF420440); and *XIAP* or *BIRC4* (U32974/U88990). (No ESTs or cDNAs have been reported for mouse *TsIAP,* despite its presence on Chromosome 7, so it was omitted from the mouse analysis.) The testis EST supporting a HERV-P-initiated human NAIP transcript has accession number DB097870. The partial rat Naip cDNA clone has accession number AF361881. Accession number U19251 refers to the human *NAIP* cDNA cloned from a fetal brain cDNA library [17,33]. The accession number of the human NAIP transcript form identified by Xu et al. [21] in the THP1 leukemic cell line is AB048534.

## Abbreviations

BIR: baculoviral IAP repeat
CAGE: cap analysis of gene expression
ERV: endogenous retrovirus
IAP: inhibition of apoptosis protein
LINE: long interspersed element
LRR: leucine rich repeat
LTR: long terminal repeat
NAIP: neuronal apoptosis inhibitory protein
NBS: nucleotide binding site
ORF: open reading frame
RACE: rapid amplification of cDNA ends
SINE: short interspersed element
TE: transposable element
TSS: transcription start site
