# Supplementary material for: Repeated Recruitment of LTR Retrotransposons as Promoters by the Anti-Apoptotic Locus NAIP during Mammalian Evolution
Source: PLoS Genet. 2007 Jan 12;3(1):e10. doi: 10.1371/journal.pgen.0030010 (PMC1781489; doi:10.1371/journal.pgen.0030010)
Supplement: Figure S1 — Cloned RT-PCR products amplified by primers specific for the two alternative LTR-derived transcripts are shown. (A) Represents RT-PCR products specific for the HERV-P-driven form (Figure 1A, form ii). The arrows show locations of primers used for quantitative real-time RT-PCR. (B) Represents products from the MER21C-associated form (Figure 1A, form iii). Recruitment of a heterogeneous ERV (5′-HAL1/LINE:AluJb/SINE-3′) was detected in sequenced clones from these isoforms. We also observed occasional exclusion of the exon from which most 5′ RACE clones were found to initiate (Figure1A, form i). These UTR variants could not be compared to those reported by Xu et al. [21] as their sequences are not available. (C) Splice variants identified by RT-PCR using primers specific for coding region exons are shown. Downstream of the first coding exon, 74 bp of a 102-bp remnant of an antisense MIRm SINE is recruited into the coding region of human NAIP in peripheral blood leukocytes. While verified by direct sequencing only in peripheral blood leukocytes, we infer transcription of this isoform in all tissues because the same band is seen in all lanes of our expression profiling experiment (Figure 4A, top band, panel O). This isoform does not preserve the established ORF (+292 to +4,503, relative to the transcript form previously reported [17,33]) and is predicted to yield a truncated protein encoding only the first and part of the second BIR domain (+292 to +888, relative to the previously reported transcript). However, downstream of the intervening MIRm SINE we report on a predicted ORF (+919 to +4,578) initiating at a start codon in-frame with the standard one (+292) that retains part of the second BIR, entire third BIR followed by the expected NBS and LRR motifs. Another minor isoform splices out the second coding exon, also disrupting the normal ORF, but utilizes an in-frame start codon to yield a novel predicted peptide (+993 to +4,412) encoding the third BIR and NBS and LRR [file pgen.0030010.sg001.ppt]

## Slide 1
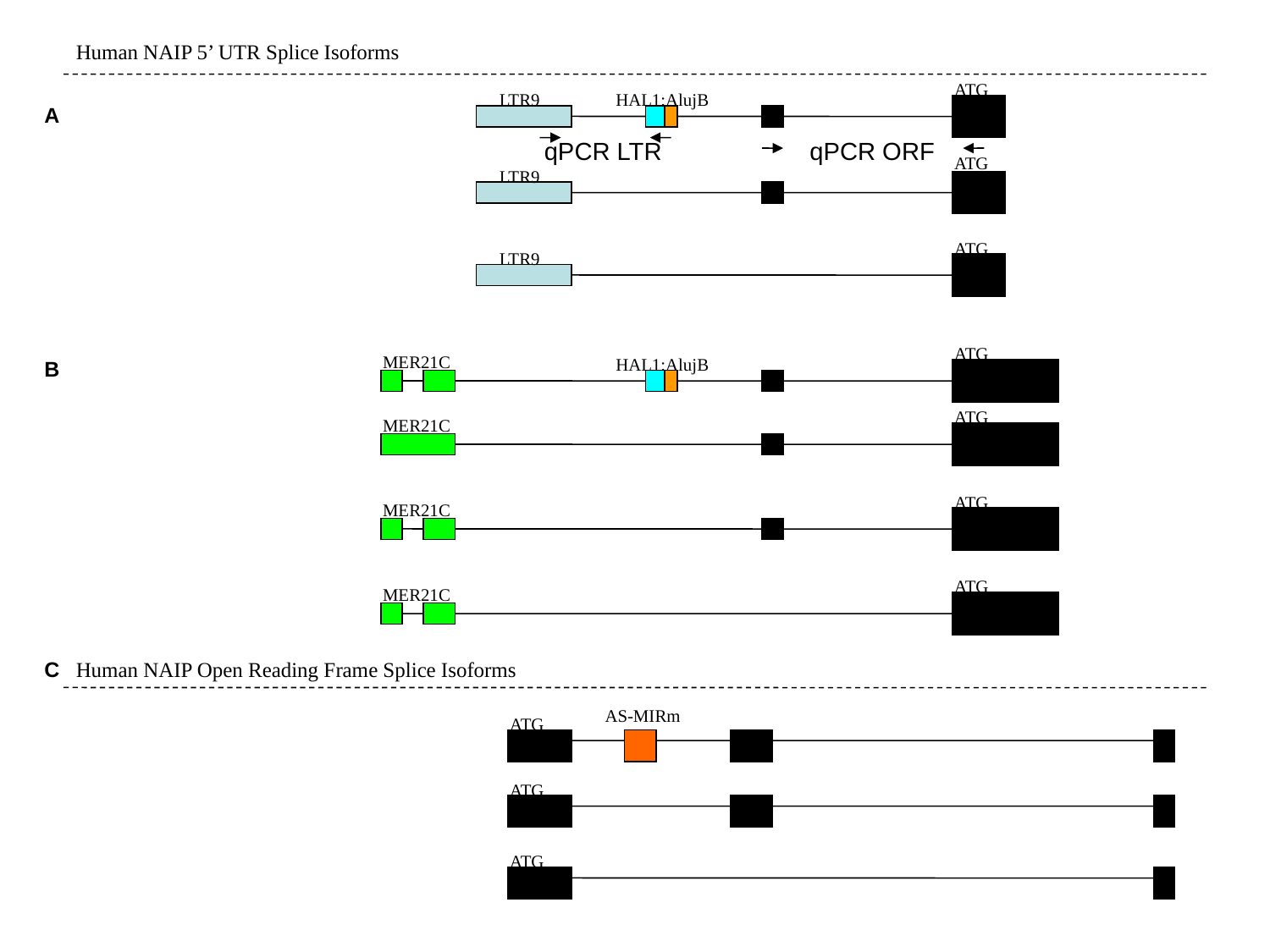

Human NAIP 5’ UTR Splice Isoforms
ATG
LTR9
HAL1:AlujB
A
qPCR LTR
qPCR ORF
ATG
LTR9
ATG
ATG
LTR9
ATG
MER21C
HAL1:AlujB
B
ATG
MER21C
ATG
MER21C
ATG
MER21C
C
Human NAIP Open Reading Frame Splice Isoforms
AS-MIRm
ATG
ATG
ATG
