# Supplementary material for: Repeated Recruitment of LTR Retrotransposons as Promoters by the Anti-Apoptotic Locus NAIP during Mammalian Evolution
Source: PLoS Genet. 2007 Jan 12;3(1):e10. doi: 10.1371/journal.pgen.0030010 (PMC1781489; doi:10.1371/journal.pgen.0030010)
Supplement: Figure S2 — (A) Cloned RT-PCR products amplified by primers specific for transcripts initiating within the ORR1E LTR are shown. Size of the ORR1E exon shows some variability among mNaip copies. Only mNaipa/b utilize a second, downstream exon within their 5′ UTRs (labeled 2). mNaipb also demonstrates recruitment of two other novel exons into its 5′ UTR, one of which utilizes partial B1F1/SINE sequence. Interestingly, we observe a mNaipe isoform that is not spliced across the length of its 5′ UTR; we are unable to comment whether it yields a functional protein, but might represent a primary transcript not yet processed by splicing machinery. (B) Splice variants for each mNaip copy using primers across coding region exons are shown. All coordinates noted below are relative to the accession numbers of the mouse Naip transcripts listed in the Accession Number section. Similar to human, we find recruitment of a repetitive exon into the mNaipa coding region, here 129 bp of the 5′ segment of a 554-bp antisense Lx LINE remnant splices in downstream of the second coding exon. This novel exon introduces an in-frame stop codon and the resulting truncated protein (+113 to +904, relative to the reported mNaipa transcript) encodes only the first two BIR domains. In addition, a novel ORF (+1,023 to +4,442) where the new initiation codon downstream of the intervening Lx LINE is in-frame with the standard one (+113) could potentially be translated to encode a protein incorporating the third BIR domain followed by the NBS and LRR. Similarly truncated proteins are expected for the isoforms of mNaipe and f which splice out the second coding exon. The C-terminal truncated peptide (+200 to +847, relative to the reported mNaipe and f transcripts) terminates within the third coding exon and is predicted to encode the first and part of the second BIR. A start codon in-frame with the standard one (+200) within the fifth coding exon yields an ORF (+892 to +4,311) that encodes the third BIR, followed by th [file pgen.0030010.sg002.ppt]

## Slide 1
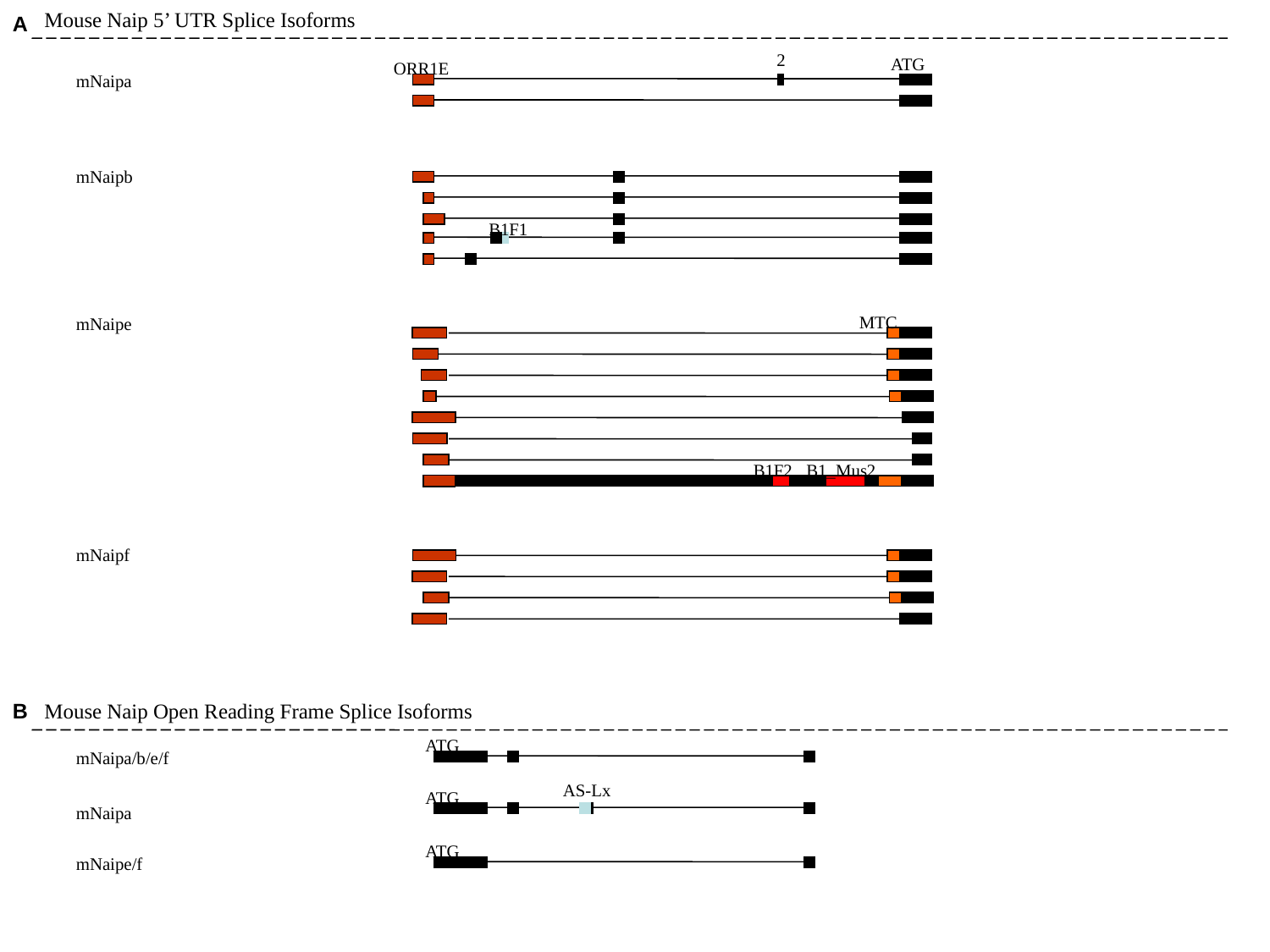

Mouse Naip 5’ UTR Splice Isoforms
A
2
ATG
ORR1E
mNaipa
mNaipb
B1F1
MTC
mNaipe
B1F2
B1_Mus2
mNaipf
B
Mouse Naip Open Reading Frame Splice Isoforms
ATG
mNaipa/b/e/f
AS-Lx
ATG
mNaipa
ATG
mNaipe/f
