# Supplementary material for: Repeated Recruitment of LTR Retrotransposons as Promoters by the Anti-Apoptotic Locus NAIP during Mammalian Evolution
Source: PLoS Genet. 2007 Jan 12;3(1):e10. doi: 10.1371/journal.pgen.0030010 (PMC1781489; doi:10.1371/journal.pgen.0030010)
Supplement: Table S1 — (29 KB DOC) [file pgen.0030010.st001.doc]

| **Table S1a.** Number and type of LTR insertions upstream (10 kb upstream + 2.5 kb 5’ gene sequence) of mouse IAP genes. | | |
| --- | --- | --- |
| **Gene Name** | **Number of LTRs** | **LTR type** |
| ***Naipa (Birc1a)*** | 7 | RMER17A2  IAPEY3_LTR  MTD (2)  MTE2b  ORR1E  RMER15 |
| ***Naipb (Birc1b)*** | 12 | ORR1E  MT2A (2)  RLTR20A/D  ORR1D1  ORR1D2  RLTR15  ORR1C1  RLTR14  IAPEY_LTR  RMER21A  ORR1B2 |
| ***cIAP1 (Birc2)*** | 4 | RMER10B  MURVY-LTR (2)  MTD |
| ***cIAP2 (Birc3)*** | 6 | ORR1C2  RLTR20A2  RMER10A  MTEa  RMER17B  MTE2a |
| ***XIAP (Birc4)*** | 1 | MT2B |
| ***Survivin (Birc5)*** | 7 | RMER4B  MTD  RMER15  MTEa  RLTR20B2/3  RMER4A/B  RMER10B |
| ***Bruce (Birc6)*** | 1 | BGLII |
| ***ML-IAP (Birc7)*** | 0 |  |

*Ts-IAP (Birc8)* was omitted from this analysis because no EST or cDNA evidence exists for its transcription, despite presence on chromosome 7.

| **Table S1b.** Number and type of LTR insertions upstream (10 kb upstream + 2.5 kb 5’ gene sequence) of human IAP genes. | | |
| --- | --- | --- |
| **Gene Name** | **Number of LTRs** | **LTR type** |
| ***Naip (Birc1)*** | 3 | MER21C  LTR9  LTR16D |
| ***cIAP1 (Birc2)*** | 3 | MER39  LTR55  MER31B |
| ***cIAP2 (Birc3)*** | 1 | LTR7 |
| ***XIAP (Birc4)*** | 2 | MER31A/B  MLT1F |
| ***Survivin (Birc5)*** | 0 |  |
| ***Bruce (Birc6)*** | 1 | MER31A |
| ***ML-IAP (Birc7)*** | 7 | MER4D1  LTR48B  LTR29  LTR2  LTR26B  LTR1  MER41A |
| ***Ts-IAP (Birc8)*** | 2 | LTR40a  LTR10E |
